# Correlations and coherence of monopolar EMG-currents of the medial gastrocnemius muscle in proximal and distal compartments

**DOI:** 10.3389/fphys.2014.00223

**Published:** 2014-06-17

**Authors:** Vinzenz von Tscharner, Christian Maurer, Benno M. Nigg

**Affiliations:** ^1^Human Performance Laboratory, University of CalgaryCalgary, AB, Canada; ^2^Move Functional, PrivateBerndorf bei Salzburg, Austria

**Keywords:** gastrocnemius muscle, current amplifier, trans-impedance amplifier, electromyography, coherence, muscle compartments, phase inversion

## Abstract

The penniform gastrocnemius muscle contains multiple heads in the proximal regions, and the aponeuroses are attached to the Achilles tendon. The multiple head structure lead to the assumption that different regions of the muscle must be activated compartment wise. The purpose of this study was to compare the correlation and coherence of EMG-currents within and between proximal and distal compartments of the medial gastrocnemius muscle, which reflect underling synchronization of motor units. It was hypothesized and shown that phase inverted signals represent a property that discriminates compartments. However, the phase inverted and non-inverted signals showed values of correlations that were indicative for highly synchronized signals. The correlation increased with the complexity of the task and was higher for the calf rising movement than while balancing in a tiptoe position. Because the muscle fibers do not span the whole length of the muscles, one has to conclude that the MUs were synchronized by synchronizing the various motor nerves. This study shows that it is essential to measure monopolar signals and use non-isometric contractions to observe synchronization of the EMG-signals. One could speculate that compartmental differences can only be observed if more complex movements that generate rotational forces at the knee or ankle are used.

## Introduction

Coherence analysis in parallel muscle fibers of fusiform muscles is usually used to measure the conduction velocity of the muscle (e.g., Barandun et al., 2009). This can be done because the electrical signal in the fibers propagates parallel to the skin. Electrodes placed on the skin have roughly the same distance to the fiber and therefore measure the same signal only shifted in time. The conduction velocity can be calculated from the time shift of the individual frequency components, which in turn can be measured by the phase angle of a coherence analysis (von Tscharner and Barandun, 2010).

The situation is different in penniform muscles. The muscle fibers are on an angle with respect to the skin surface. Therefore also the electrical field that propagates along the muscle fiber has this inclination with respect to the skin surface. Electrodes placed on the skin will therefore record a potential predicted by model computation that predominantly reflects the potential generated by the part of the fiber that is closer to the upper aponeuroses (Mesin et al., 2011).

Thus if the electrodes mounted on the skin above a penniform muscle are sufficiently separated, the signal measured at the electrode results from different muscles fibers. Any coherence and phase angle of a monopolar EMG-signal is therefore related to a kind of time synchronized activation of different muscle fibers, rather than a time delay of the electrical signal of the same muscle fibers. Furthermore, the classical view that a bipolar EMG signal yields a good approximation to the derivative motor unit action potential (MUAP) is not anymore valid in penniform muscles. It seems therefore advisable to use monopolar EMG measurements on penniform muscles.

The medial gastrocnemius muscle is a penniform muscle with a well-defined anatomical structure. In the proximal region there is a medial head, an intermedio, and intermedio-lateral head (Ashaolu et al., 2013). The fascicles are attached to the upper and lower aponeuroses thus they form a penniform structure with a large, force dependent pennation angle that covers the range between 15 and almost 45° of the fascicles (Muramatsu et al., 2002). In rat gastrocnemius endplates of distal fibers are located on the proximal third of their lengths. Endplates of intermediate fibers are located at half fiber length (Dekhuijzen et al., 1986). The same location is assumed for human gastrocnemius muscle (Vieira et al., 2011). It is assumed that a similar, not absolutely centered distribution of locations of endplates is characteristic for the human gastrocnemius muscle, which affects the timing of the extinction signals on both ends of the muscle fiber (Dimitrova et al., 1999, 2002).

When muscles exert a force one differentiates isometric contractions and task specific contractions, for instance balance, walking, and running tasks. While running, muscular event starts at some point in time and end after a while e.g., 150 ms. The EMG intensity shows a distinct, periodic modulation during a muscular event, which represents the Piper rhythm (Maurer et al., 2013). To generate such a modulation, the motor unit action potentials (MUAP) must arrive at the right location at almost the same time. The current understanding of motor unit (MU) recruitment is primarily based on measurements made during isometric contractions. The MU recruitment during a dynamic task may be more tuned toward recruitment of fiber types and MUs that are necessary to perform a specific task (Hodson-Tole and Wakeling, 2009). To perform a certain task, it may be important to selectively activate certain muscle compartments. However, instead of muscle compartments one could also think of activating task groups of MUs that fulfill the demands required during different movements (Loeb, 1985). Studies that address this kind of questions cannot be performed at low muscle activity. At low muscle activity an automatic decomposition of intramuscular MU is possible (Vieira et al., 2010). At high muscle activity this method may not be very reliable, and a more macroscopic approach has to be applied. It has been shown that for a cycling movement the EMG-intensity was highly correlated within the compartments of the medial gastrocnemius muscle (0.56 < *r*^2^ < 0.62). However, contrary to the expectation, the raw EMG-signals were not correlated (Wakeling, 2009). The reason for this may be that synchronized signals were eliminated from the measured EMG-signal by the common mode rejection of the bipolar amplifiers (von Tscharner et al., 2013). Thus monopolar measurements are necessary if one wants to quantify the correlation of raw EMG-signals during a dynamic task (von Tscharner, 2014).

In addition to the measurements that were used to compare EMG-potentials and EMG-currents (von Tscharner et al., 2013) a new set of experiments was made using current amplifiers only to study the properties of the activation of the medial gastrocnemius muscle. The first findings revealed a task dependent, in phase correlation, which was interpreted as a result of synchronization of motor units of the medial gastrocnemius muscle (von Tscharner, 2014). This synchronization resulted in a strong, in phase correlation of signals from adjacent electrodes and a weaker, reverse phase correlation. The ratio of in phase to reverse phase correlation increased with the complexity of the movement. However, additional hypothesis regarding the correlation between compartments had to be tested. A limitation of the previous work was that it could not reveal whether the in phase correlation was a frequency independent mirror image. Such an interpretation seemed too simple and would not show a frequency dependency that may be caused by the way the motor units synchronize.

The purpose of this study was to compare the correlation and coherence within and between muscle compartments. Specifically the coherence had to be addressed to assess whether there was a frequency dependency of the correlation or phase inversion. The medial gastrocnemius muscle was selected for this purpose because the results can be compared with published results where raw, bipolar EMG-signals were uncorrelated, although the EMG-intensities were correlated (Wakeling, 2009). Furthermore, inspection of Figure 5 of the article Hodson-Tole et al. (2013), channel 12 and 14 show phase inverted signals that were not discussed. In previous work such phase inversions (mirrored signals) were observed as outliers in the proximal compartment of the medial gastrocnemius muscle (von Tscharner, 2014). It was therefore hypothesized that these phase inverted signals were not outliers but represent a new property that reveals aspects caused by MU recruitment. It was speculated that phase inversion may be a property that characterizes muscle compartments or, may be, characterizes task groups.

## Materials and methods

### Subjects and tasks

Six female and seven male subjects were recruited among colleagues of our research team. They were between 20 and 30 years of age and mostly well trained recreational athletes. Conjoint Health Research Ethics Board of the University of Calgary has approved the measurements on the gastrocnemius muscle. All experimental procedures were carried out in accordance with the principles of the Declaration of Helsinki. The subjects performed first a repetitive vertical calf rising movement e.g., a plantar flexion of the ankle joint while standing on the forefoot followed by relaxing back to a horizontal position. The up and down movement was performed at a self-selected repetition rate while standing on the edge of a step for 70 s. After 1 min rest, the subjects stood quietly for 70 s in a tiptoe position (maximal calf rising position) while standing on the same edge of a step.

### Recording of EMG signals

The monopolar EMG-currents (nA) were measured with a trans-impedance amplifier (bandwidth 10–500 Hz) as described previously (von Tscharner et al., 2013). The trans-impedance amplifier measures the current that has to be extracted or injected into the surface electrodes (Ag/AgCl, Norotrode dual electrodes, Myotronics-Noromed Inc., Kent, WA, US) to keep the electrode at ground or reference potential. Thus all electrodes remain permanently at ground potential and therefore no lateral currents can occur.

**Figure 1:**
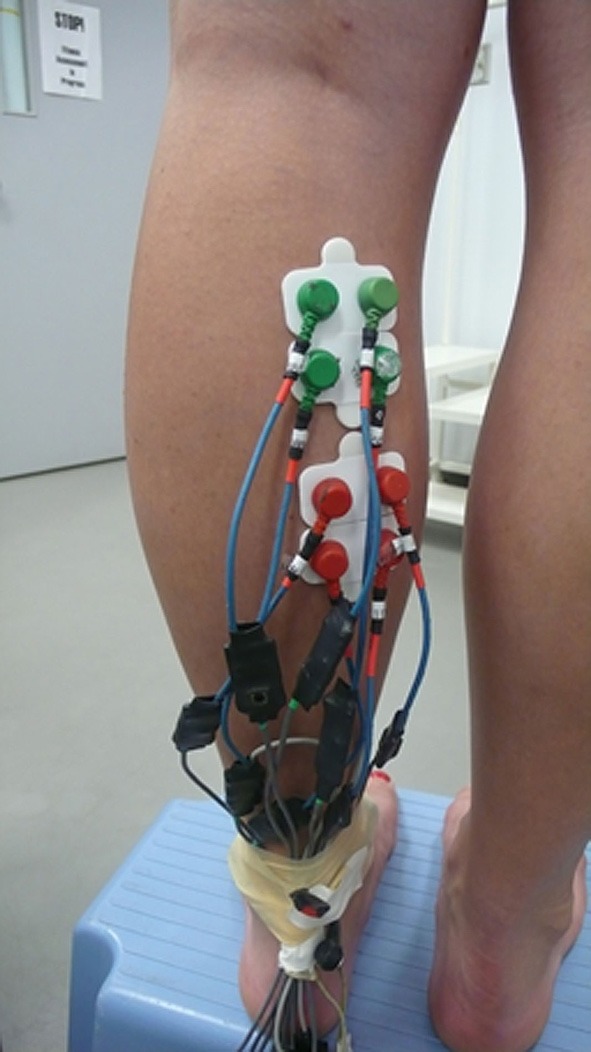
**Picture of electrode placement on the medial gastrocnemius muscle**.

The ground electrodes, two of them, were placed on malleolus medialis and lateralis. It is important to have a high capacity ground level because all measured currents flow in and out of this ground site. Four measuring electrodes were placed in the proximal compartment of the medial gastrocnemius muscle. The exact location was first selected from ultrasound images of the muscle. Later on the location was palpated. The location of electrode #1 was 30 mm below the fossa popliteal and about 20 mm medial of the line separating the medial and lateral gastrocnemius. In this location the muscle fibers have a distinctly visible pennation angle and muscle structure seems to be well-defined. The second electrode was 21 mm medial to the first one. The third and the fourth electrodes were arranged distal to electrodes one and two, in the direction of the muscle fibers (Figure 1).

Another four electrodes were placed in the distal compartment of the medial gastrocnemius that previously showed simultaneous muscle activation (English et al., 1993; Vieira et al., 2010; von Tscharner et al., 2013). The four electrodes (electrode #5 to electrode #8) were positioned proximal to the line where the inner- and upper aponeuroses merge, about 4 cm below the lower electrodes of the proximal compartment. In this location the pennation angle gradually decreased and was less than in the proximal compartment. Thus the eight electrodes formed a square on each, the proximal, and the distal compartments of the medial gastrocnemius. The electrodes were mounted to the skin surface after shaving off the hairs and rubbing the surface with alcohol pads for hygienic reasons, however, if allowed, cleaning with water may be more appropriate (Merletti and Parker, 2004, accessible via internet). From the recordings, a signal encompassing 60,000 points (25 s) was selected for the analysis. The signal was filtered using a wavelet based low pass filter starting at *cf* = 300 Hz with a 3 db point at 425 Hz (von Tscharner et al., 2013). The 60 Hz (40 points per cycle) line frequency contamination was extracted from the signal by a line frequency averaging method (von Tscharner et al., 2013).

### Correlation analysis

A correlation analysis was done between the EMG signals of electrodes within a compartment and across compartments. This study focuses on the correlation between the EMG, and a detailed analysis of the amplitudes was not performed. The analysis of correlation was the simplest measure of similarity between the EMG signals. A correlation was deemed not significant if it was smaller than the maximum correlation obtained by time shifting the two signals by an arbitrarily, but sufficiently long time delay (200 ms) with respect to one another. A sufficiently long time delay was selected based on previous work, which showed that muscular events are of the order of maximally 100 ms (von Tscharner et al., 2003). One also knows that synchronization of motor units are necessary to form the Piper rhythm in EMG signals which is higher than 20 Hz (period 50 ms) (Maurer et al., 2013). It is therefore reasonable to select a time longer than 100 ms. Various delays were tested to find out that a 200 ms delay was sufficient to yield a minimal coherence.

### Coherence analysis

Coherence analysis, which is performed in frequency space applying the Fourier transform, is a well-known method to compute the frequency dependent relationship (correlation) between two signals. (Rosenberg et al., 1989). From the eight EMG channels *i* ∈ [1..8] one channel was selected as the source (s), and one channel was chosen for the response (r). The definition of source and response is arbitrary in this study, however, can be important for the interpretation of the phase angle as one can determine, which of the two signals arrives first at the electrode. The coherence analysis was done in the Fourier domain. The EMG − signal_*i*_ (*t*) was subdivided into sequences of 512 data points. With the sampling frequency of 2400 Hz one achieved a frequency resolution of the coherence analyses of 4.7 Hz. This was deemed sufficient to observe changes that occur in the frequency range of interest for EMG signals (10–500 Hz). For every sequence (n) the Fourier transform was calculated, leading to the frequency spectrum Fs_*n*_ (λ) for the source and Fr_*n*_ (λ) for the response. The absolute value (coherence) and the phase angle (Φ) of the mean value of the product of source Fs_*n*_ (λ) and response Fr_*n*_ (λ) was calculated:

$$\text{coherence}\left(\lambda \right)\;=\frac{{\left|\text{mean}\left({\text{Fs}}_{n}\left(\lambda \right)\cdot {\text{Fr}}_{n}{\left(\lambda \right)}^{*}\right)\right|}^{2}}{\text{mean}\left({\text{Fs}}_{n}\left(\lambda \right)\cdot {\text{Fs}}_{n}{\left(\lambda \right)}^{*}\right)\cdot \text{mean}\left({\text{Fs}}_{n}\left(\lambda \right)\cdot {\text{Fr}}_{n}{\left(\lambda \right)}^{*}\right)},$$

$$\phi \left(\lambda \right)\;=\text{arg}\left(\text{mean}\left({\text{Fs}}_{n}\left(\lambda \right)\cdot {\text{Fr}}_{n}{\left(\lambda \right)}^{*}\right)\right)$$

^*^ indicates the conjugate complex value. The mean is taken across all sequences *n*.

The phase angle varies between −π and +π and thus flips between the two values when it exceeds one of them. The Matlab function “unwrap” was used to prevent the flipping.

A time shift between the source and the response signal generates a slope in a graph of the phase angle. The slope is 2π/(sampling frequency) if the signals were time shifted by one sample. This corresponds to a slope of 1/400 rad/Hz.

## Results

### Within compartment correlations

**Figure 2:**
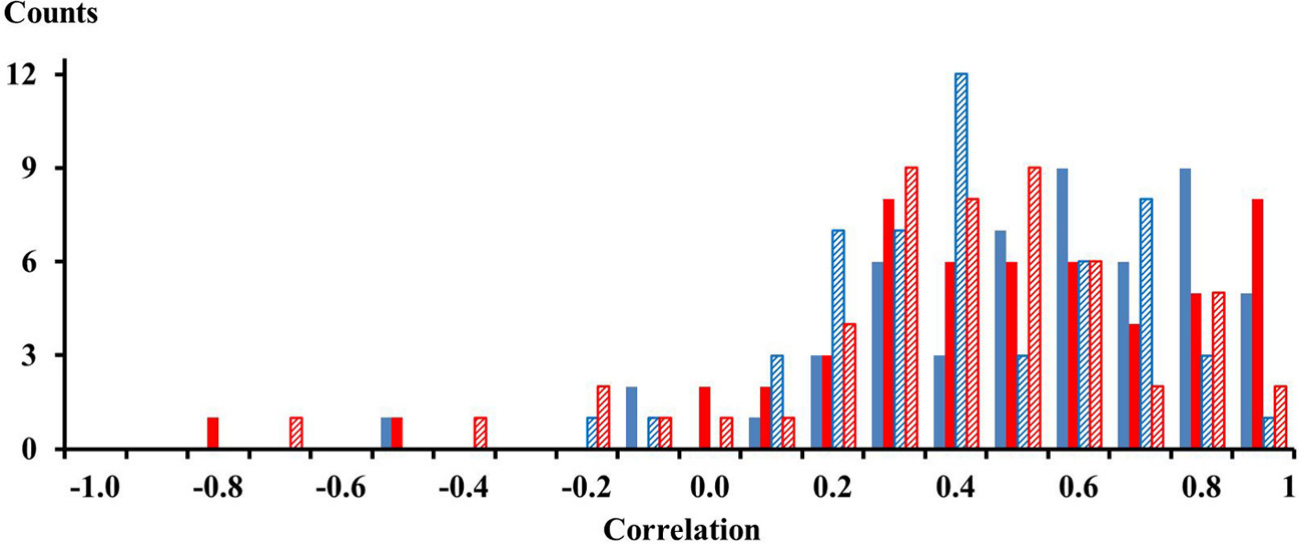
**Solid blue and red bars represent correlations among neighboring electrodes of the proximal and distal compartment, respectively, observed while performing a repetitive calf rising movement**. Dashed blue and red bars represent correlations among neighboring electrodes of the proximal and distal compartment, respectively, observed while standing on the forefoot in a tiptoe position. There were four combinations for each compartment and subject, thus 52 correlations per color. The numbers on the *x*-axes represent the lower edge of the bins.

For each of the 13 subjects there were 4 pairs of electrodes with an inter electrode distance of 21 mm thus yielding 52 correlations for one compartment. For two compartments and for two conditions, the calf rising and the tiptoe positions, there were in total 208 correlations. These correlations are shown in the histogram of Figure 2. Out of the 208 correlations there were only 10 that had negative values. On average, the mean correlation of neighboring electrodes on the proximal compartment was 0.48 (STE 0.04) and 0.43 (STE 0.04) for electrodes on the distal compartment while calf rising. There was no difference of the correlation between electrodes in the medial lateral directions and those aligned along the muscle fibers. Thus signals are, according to a binomial test, significantly correlated within the proximal and distal compartments.

### Correlations between compartments

**Figure 3:**
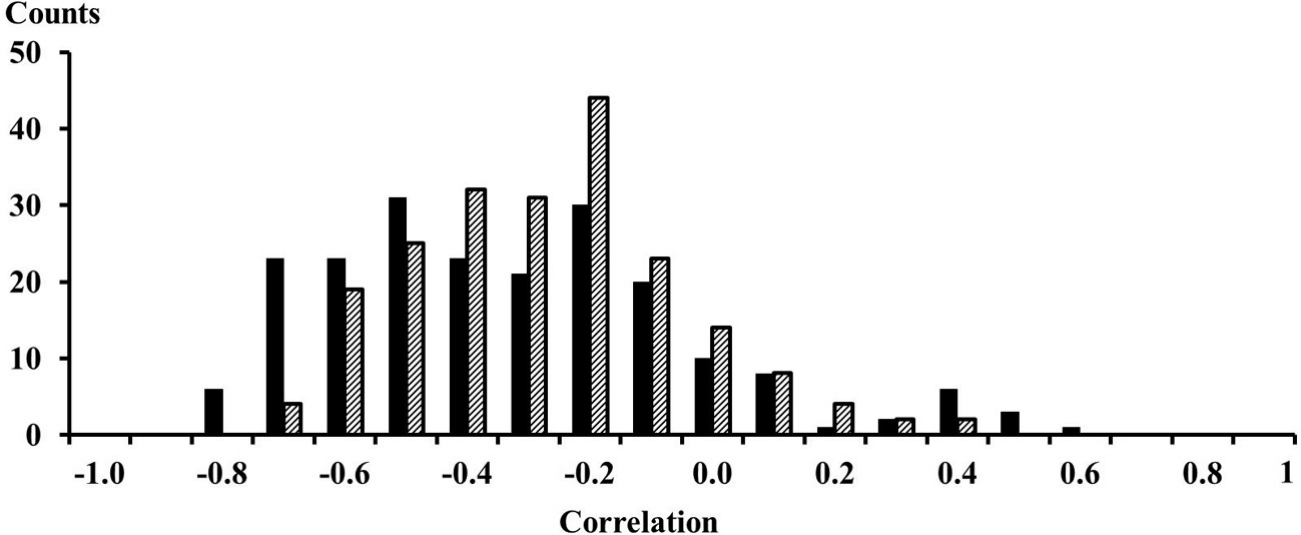
**Histogram of the 208 correlations between EMG signals from proximal and distal electrodes**. Solid black bars were observed for the calf rising movement; dashed bars represent results obtained while standing on the forefoot in a tiptoe position. The numbers on the *x*-axes represent the lower edge of the bins.

**Figure 4:**
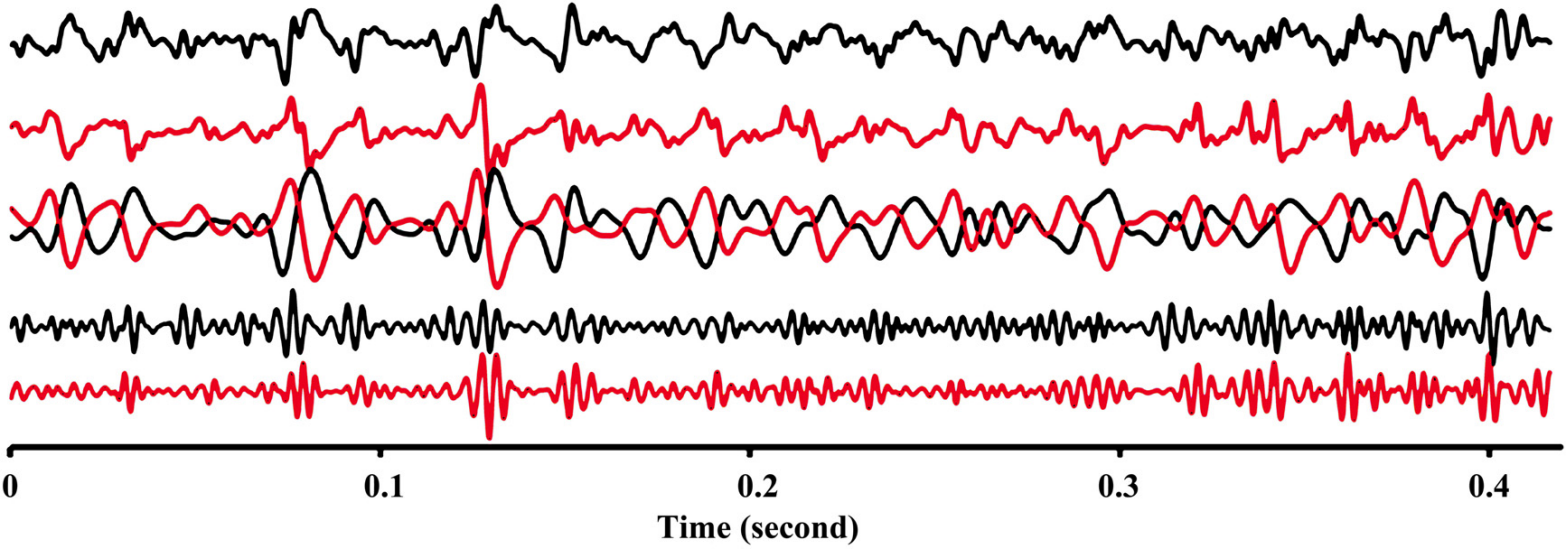
**High resolution display of raw EMG-currents (arbitrary units) of electrode #3 of the proximal compartment (black) and of electrode #7 of the distal compartment (brown) during a calf rising movement**. Top two lines represent the measured raw signals. The overlapping middle two lines represent low pass filtered (3 dB point at 140 Hz) EMG-currents multiplied by 2. These lines reveal the phase inversion. The lowest two lines represent the high pass filtered (3 dB point at 180 Hz) EMG currents.

The EMG signals between signals from proximal and distal electrode pairs were predominantly negatively correlated indicating that there is a phase inversion between signals from the proximal and distal compartment of the muscle (Figure 3). A high resolution display of the EMG-currents of one subject reveals this phase inversion (Figure 4). The low pass filtered EMG-currents showed the phase inversion distinctly. The high pass filtered EMG-currents did reveal some correlations but the average phase relation cannot visually be assessed. Only 61 out of 416 correlations between compartments were positively correlated. On average the correlations were −0.375 (STE 0.021) for the calf rising movement and −0.321 (STE 0.015) when standing in a tiptoe position. Thus in contrast to within compartments, where the correlations were positive, the correlations between the proximal and the distal compartments were, according to a binomial test, significant but negative.

### Decrease in correlation when standing in a tiptoe position

**Figure 5:**
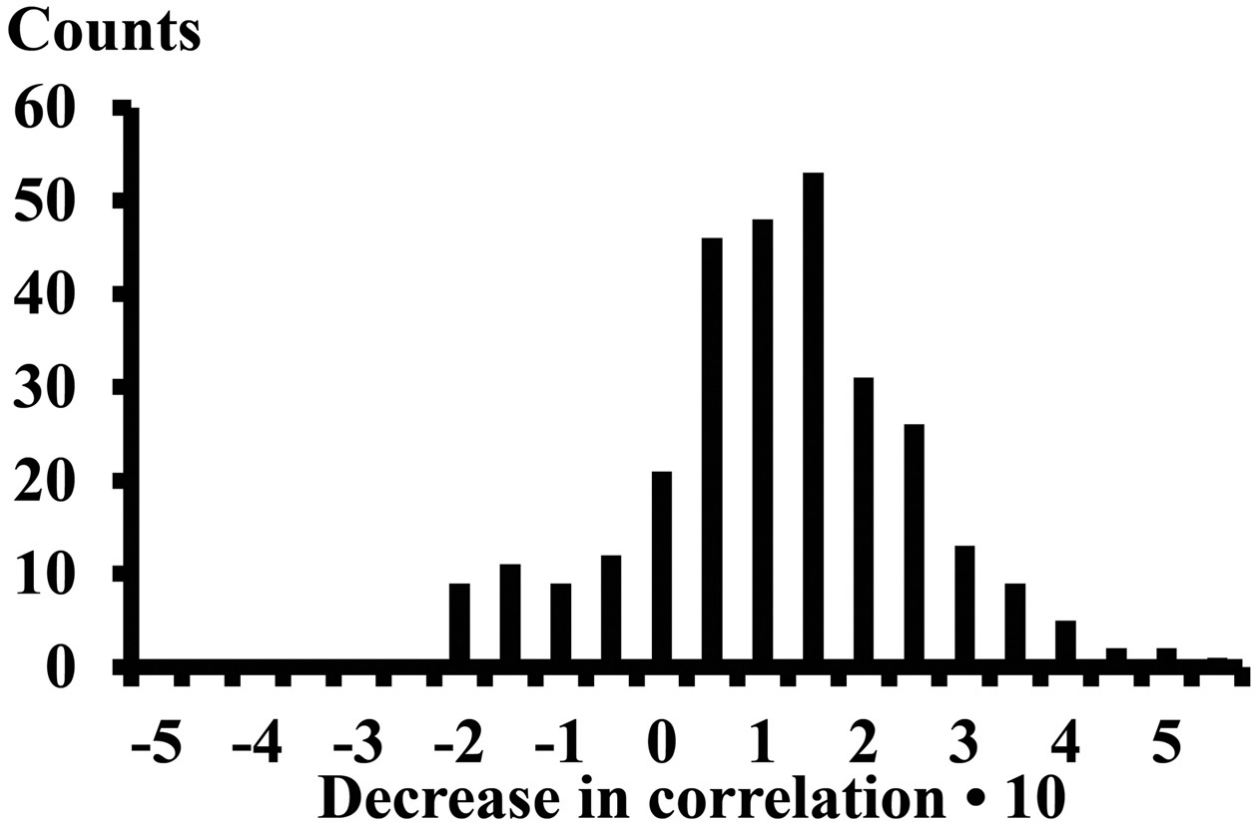
**Decrease of the correlation when changing from the calf rising movement to standing on the forefoot in a tiptoe position**. Result of 312 cases. The numbers on the *x*-axes represent the lower edge of the bins.

The differences of the correlations (13 subjects, 4 electrode pairs in each compartment, and 16 inter compartment electrode pairs, thus 312 cases) observed while performing the calf rising movement or standing on the forefoot was on average 0.089 (STE 0.008) (Figure 5). The correlations decreased when changing from calf rising to standing in a tiptoe position in 257 cases, increased in 41 cases and the signum changed in 14 cases.

### General observations

All correlations for cases that did not follow the general trend were statistically significant. Therefore they represent variations that are subject specific, and one has to look at individual results to obtain a subject specific knowledge of the interplay of compartments. Frequently, the EMG of electrode #4 was visibly less correlated to the EMG recorded from other electrodes. It seemed that this electrode was placed on the boundary of the compartment. However, the results were included in the above analysis. Furthermore, electrodes #1 to #3 of the upper compartment yielded less amplitude fluctuations than the electrodes in the lower compartments. Visual inspection of some data showed that the low frequency components were correlated, however, data of other subjects seemed to indicate that higher frequencies contributed to the correlation. Therefore, relevant aspects of EMG signal correlation are to be found in frequency space. A coherence analysis in frequency space yielded the results presented and discussed below.

### Coherence analysis

**Figure 6:**
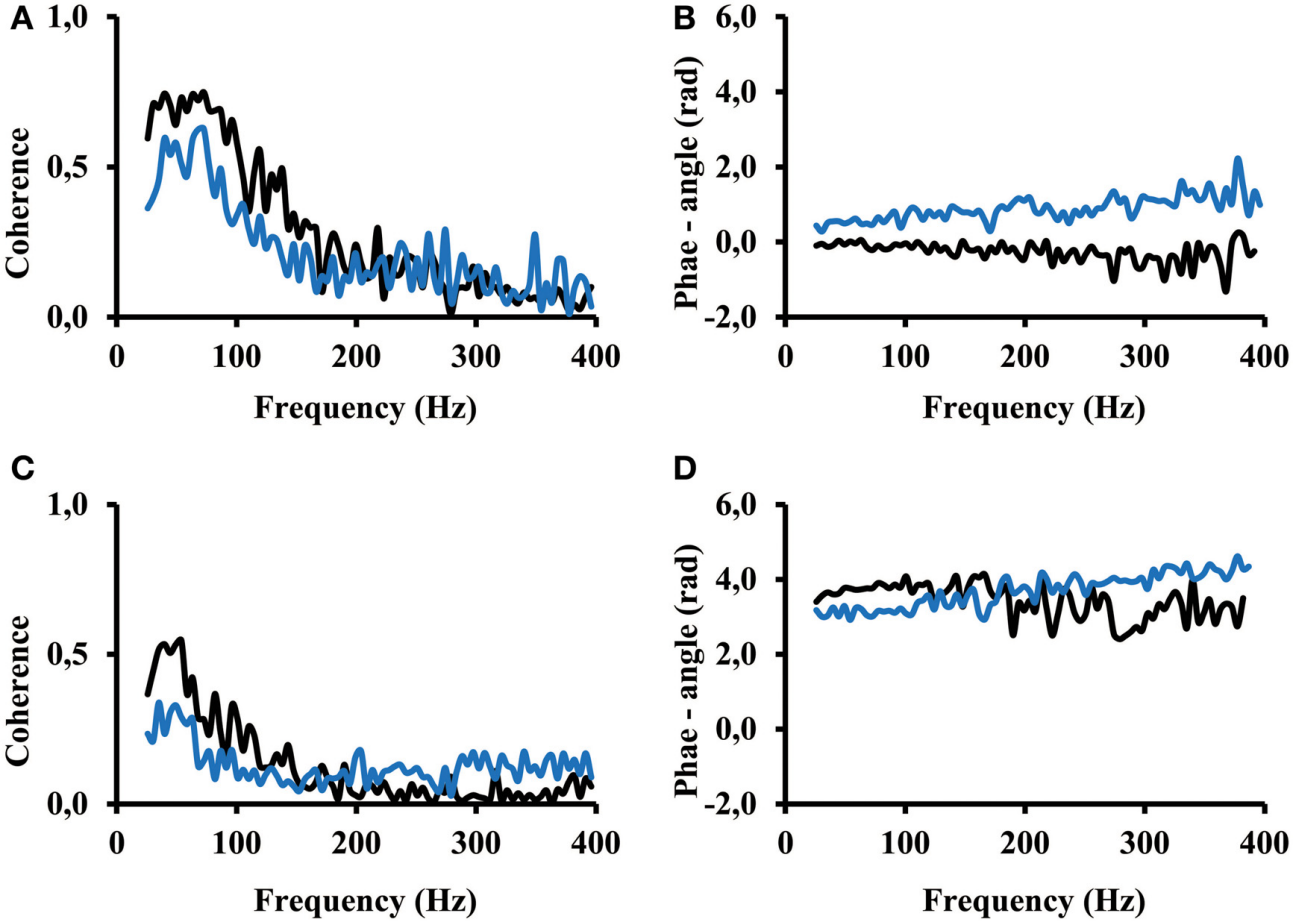
**(A)** and **(B)** Coherence and phase angle within the proximal compartment (electrode #1 vs. electrode #2 (black) thus in medial lateral direction and electrode #1 vs. electrode #3 (blue) thus along the direction of the muscle fibers. **(C)** and **(D)** Coherence and phase angle between the proximal and distal compartment (electrode #1 of the proximal compartment vs. electrode #6 (black) and electrode #8(blue) of the distal compartment. Coherence below 0.09 is coherence that can be obtained by 200 ms time shifted signals, thus by uncorrelated signals.

Seven out of the 13 subjects showed a coherence result similar to the result of subject 12 (Figure 6). An arbitrary selection of electrodes was used to show representative result. One can see that for both, the calf rising movement and the standing in a tiptoe position, significant coherence occurs in the frequency range from 25 Hz to about 200 Hz. Coherence was not zero but smaller for higher frequencies. The fact that the phase angle remains the same at low and high frequencies indicates that even the coherence at frequencies above 200 Hz contributed to the overall correlation. The coherence is distinctly lower while standing in a tiptoe position compared to the one observed for the calf rising movement (Figures 6A,C). This finding confirms the finding of the correlation analysis shown above (Figure 5).

The phase angle was in most cases around zero rad for electrodes of the same compartment (Figure 6B). In contrast, phase angle was around π rad between signals from the proximal and the distal compartments (Figure 6C). A slope of 1/400 rad/Hz corresponds to a time shift of 0.4 ms between the two EMGs recorded from two separate electrodes. In all cases, the slope computed between 25 and 400 Hz was below 2/400 rad/Hz, thus indicating that the time shift between two EMGs, whether within or across compartments, was always below 0.8 ms (time of two sampling points).

**Figure 7:**
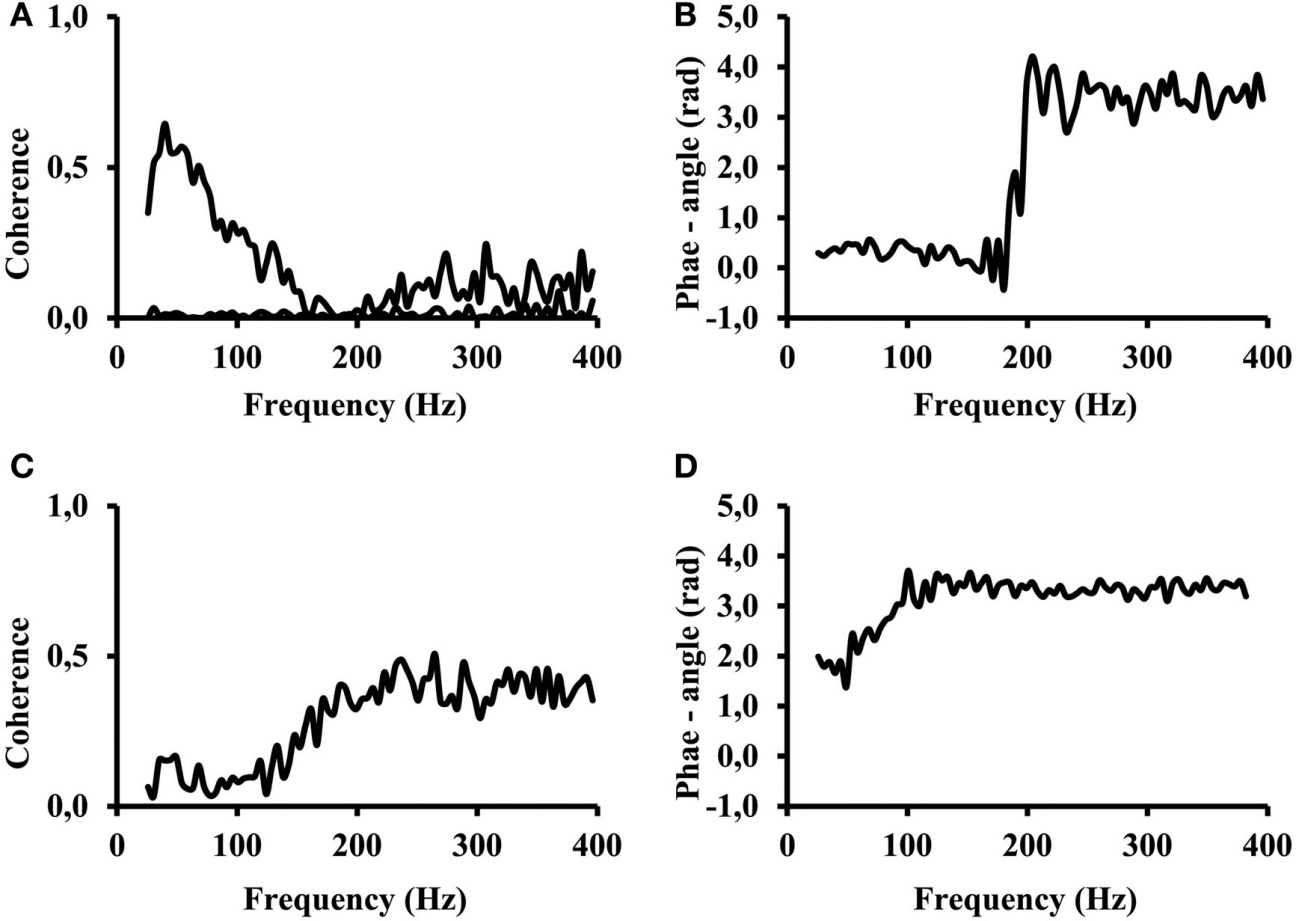
**(A)** Coherence while calf rising of a subject with distinct signal in high and low frequency bands electrode #1 of proximal compartment vs. electrode #6 of distal compartment (black). Coherence when the signal of electrode #1 was shifted by 200 ms; this represents the coherence of non-correlated signals (black line on bottom). **(B)** phase angle for the coherence shown in **(A)**. **(C)** Coherence while calf rising of electrode #1 vs. electrode #6 for a subject where coherence at high frequencies is larger than at low frequencies. **(D)** phase angle of the coherence shown in **(C)**.

However, there were signals that revealed aspects where our ability to interpret them was limited. The signals of certain subjects revealed a different phase angle at low and high frequencies of the coherence spectrum. The coherence in many cases showed a first band in the frequency range between 25 Hz and about 200 Hz and a second, high frequency band, at frequencies above 200 Hz. Two special cases taken from calf rising movements revealed the effect of two frequency bands most distinctly (Figure 7). The coherence of one subject showed a distinct low frequency band, and the coherence almost disappeared at 200 Hz (Figure 7A top line). This band was followed by a larger coherence in a second frequency band that covered all higher frequencies between 200 and 400 Hz. A second subject showed a very low coherence between electrode #1 vs. electrode #6 at low frequencies and high coherence in the high frequency band (Figure 7C). The coherence in the low and high frequency band was significantly larger than the reference coherence of non-correlated signals (Figure 7A, bottom line). One can observe that the coherence within the two frequency bands do not necessarily have the same phase angle. In terms of correlations this means, the signal at lower frequencies were positively correlated, whereas the higher frequencies were negatively correlated. In these two cases only the coherence measured at high frequency reflected the phase inversion that was otherwise typical for electrode pairs that belonged to the proximal and distal muscle compartments.

The result clearly indicates that coherence below and above 200 Hz has a different origin. Although most subjects used both frequency bands in parallel, certain subjects were able to selectively control the activity in the two bands.

## Discussion

It was hypothesized that the synchronization of MUs leads to correlated and coherent raw monopolar EMG-currents. The purpose of this study was to compare the correlation and coherence of the raw EMG-current signals within and between muscle compartments. In summary, it was shown that the correlations among EMG-currents recorded by electrodes within a muscle compartment were positively correlated whereas a negative correlation was usually observed for electrode pairs between compartments. This finding was confirmed for both, the calf rising movement and balancing in a tiptoe position. However, the coherence analysis showed that there was a different weight of the high and low frequency coherence for the two conditions. This finding is not primarily related to the compartments and requires further research. The characteristic feature of a negative correlation that discriminated the two compartments is a new finding that shows that at least part of the signal is phase inverted and not time shifted. Phase inverted means that the |phase angle| = π or in other words the polarity of one signal was reversed with respect to the other signal. These aspects will be discussed in the next paragraphs.

### Phase inversion

EMG-signals are viewed as superposition of motor unit action potentials (MUAP) or in the present case as motor unit action currents (MUAC). Two discharge processes are elicited at the endplates of the motor-neuron; in one process the ion currents propagates toward the lower aponeuroses in the other toward the upper aponeuroses, thus in opposite directions along the muscle fiber. The MUAP and MUAC, when measured at the surface of the skin, reflects the interference of the depolarization processes of multiple muscle fibers. A volume conductor model computation of the MUAP showed that the signals expected from a fusiform muscle and from a pinnate muscle are very different (Mesin et al., 2011). In a pinnate muscle only the depolarization wave that was produced by the part of the fiber that is closer to the upper aponeuroses, and the extinction at the end of the fiber, produce a noticeable potential fluctuation at the surface of the skin. This potential does not propagate along the surface of the skin; in contrast, it appears and disappears in a small local area. The appearance in another, sufficiently distant area is only correlated if the muscle fibers were activated at the same time. Let's assume that only one such area is activated and one measures in a differential mode with a linear array of electrodes. In that case one can foresee that if the first electrode of a bipolar setup is on that site the signal has a positive polarity whereas if the second electrode is on that site the signal has a negative polarity. Thus one can see a phase inversion. This might be a possible explanation for the phase inversion seen in Figure 5 of Vieira et al. (2011). However, this cannot be the explanation for the phase inversion seen in monopolar EMG-current measurements.

The depolarization of a muscle fiber always starts with a sodium influx. Thus the current always has to start by a current from the electrode to the body. For the time being there is no model computation that would account for a phase inversion. A slow drifting current was observed while the amplifiers were developed and was compensated by a feedback current (high pass filter) (von Tscharner et al., 2013). This current was primarily thought to be caused by the electrochemical potential generated at the interface between the electrode and the skin. These currents do not have the possibility to produce a phase inversion. As an alternative one could speculate that there are some unknown compensating DC currents in the tissue surrounding the fascicles. The currents generated by the depolarization of the fibers could then be mirrored, and thus the measured currents would be phase inverted. A possible reason for the phase inversion could be that nature wants the muscle as a whole to remain isoelectric. A distant location, far away from the muscle, would then not see the synchronized activation of the muscle. Up to now it was not possible to gain additional information on such currents or to model a phase inversion without excessive speculations.

### The interpretation of correlation and coherence

Correlation or coherence between the EMG recorded by two electrodes can have multiple reasons. The three dominant ones are (a) cross talk, (b) signal from the same MU, and (c) synchronized signal from different MU. (a) Cross talk can be exclude because current measurements allow no lateral currents and a crosstalk between very distant electrodes e.g., electrode #1 and electrode #8 is very unlikely to be of the same order of magnitude than crosstalk between adjacent electrodes. (b) The signals belong to the same MU. This means there is a common motor nerve activating the different muscle fibers. It is unlikely that the signals of all eight electrodes were controlled by a single motor nerve. (c) The motor neurons of the different MUs are synchronized. This is the most likely explanation for a common shape of the absolute EMG signals. However, this explanation is based on an interpretation of the data and there was no independent measure of the synchronization to validate the findings.

The results showed that the mean absolute correlations within a compartment (*r* = 0.48) was higher than the mean absolute correlation across compartments (*r* = 0.375). Thus the mean correlation was 0.105 (STD 0.043) correlation units higher within a compartment than across the compartments. One does expect that the signal from one MU has a high correlation, as the action potential is released to all fibers of this motor unit at the same time. Measurements performed within a small area should, therefore, have a high correlation as they record predominantly the signal from the same MUs. However, in our experiments we could observe a high correlation between very distant locations. In fact, on average, the correlation between the distal electrodes was only 0.105 smaller than the correlation between neighboring electrodes. The difference is too small to be confident that within a compartment the common MUs were the dominant reason for the correlation. The fact that signals from very distant positions, signals from the proximal and distal area, were correlated indicated that the synchronization of the motor nerves played an important role.

Our results showed a decrease in the correlation when changing the motor task from calf rising to standing in the tiptoe position. One might argue that the first motor task requires more force and, therefore, larger motor neurons are activated (size principle). However, in previous experiments (von Tscharner et al., 2013) it was shown that for a guided isometric contraction at 40% of maximal voluntary contraction correlations were very low (<0.2) and below the statistical limit at higher frequencies. The later experiment was performed on a Biodex machine, where subjects were strapped into a fixed position and for the movement no control of balance was necessary. The results therefore seem to indicate that force might not be the important factor for the correlated activation of multiple neurons, rather that the complexity of the task be the critical factor.

It is known that MUAP and also MUAC have characteristic shapes. Within the spectrum of MUAP the power at low frequencies are indicative for the presence of a MUAC whereas the power at higher frequencies are more likely representative for the fine structure of the MUAC. Therefore the low frequency components of the EMG are more likely to show a high coherence than the high frequency components. This was confirmed by the present results where the highest coherence was in most cases within the frequency range of 25–100 Hz (Figure 6). The odd exemption (Figure 7C) might be possible but has either to be seen as an outlier compared to the other coherence spectra of the same subject or it might be part of a task group. However, it might be this kind of a signal that indicates that a specific MU was used for a different purpose and the signals did not correlate with all other signals. These are the kind of signals one has to look for in finding out the functions of different MUs. Although the highest coherence was found at low frequencies, the coherence above 200 Hz were significantly higher than the coherence from signals that were 200 ms time shifted (Figure 7A, bottom trace). At least, the coherence analysis showed that there were two frequency bands and that the coherence in these two bands were not generated by the same physiological processes, they showed some independency from one another. In summary it seems that the correlation and larger coherence are predominantly caused by low frequency components of synchronized MUs.

### Correlations within a muscular compartment

The results clearly showed that the correlations between raw EMG-currents within one muscle compartment were mostly positively correlated. Repetitive calf rising requires a more complex neuromuscular control than balancing in a tiptoe position and requires a higher degree of synchronization of MUs, which corresponds to larger correlation values (Figure 5). The effect of the complexity on the correlation was previously investigated systematically (von Tscharner, 2014). As shown in this study, it also leads to higher coherence values especially in the low frequency range of 25–100 Hz. However, coherence remains above the level of uncorrelated signals up to 400 Hz (Figure 6A). Furthermore, balancing in a tiptoe position requires a higher neuromuscular control than a simple isometric contraction on a Biodex machine, but the present and previous measurements showed that coherence was low, but remained significantly above zero for both tasks (von Tscharner et al., 2013).

It was shown recently that measurements of EMG-currents have a finer spatial resolution than measurements of EMG-potentials which becomes important when the targeted compartment is small or if one reaches the edge of the compartment (von Tscharner et al., 2013). In the present study there were occasional negative correlations within the proximal and within the distal compartments. These phase inversions happened between electrodes that were only 21 mm apart. One has therefore to realize that the territory of negatively correlated signals may be as small as or smaller than the currently used inter electrode distances.

### Correlations between muscular compartments

A negative correlation between EMG signals recorded at different places was observed in many pilot studies. The systematic investigation of the correlations and coherence between a proximal and distal area on the medial gastrocnemius confirmed that in a significant majority of cases, the correlations between electrodes placed on these two compartments were negative, but the absolute values of the correlations were highly significant. It seems that the two areas are activated by a synchronized neurological control. It is obvious that when raw EMG-currents are correlated then, in turn, total intensity is also correlated. The correlation of total intensity (square root of EMG-power) has been shown previously; however, the correlation of the raw EMG-signal was not observed (Wakeling, 2009). The most likely reason for not observing a correlation in the raw EMG-potential was the common mode rejection that eliminates exactly the in phase part of the signal (von Tscharner, 2014). The reverse phase, although it does not represent the whole power of the EMG-signal, is sufficient to detect the time dependent increase in muscle activity and elicit correlated EMG-power. The present analysis was limited to eight electrodes and could therefore not precisely delineate the geometrical borders of the compartment. An automatic segmentation of surface EMG images revealed less spatial resolution but clearer indication of muscle compartments (Vieira et al., 2011). One could be inclined to assign the locations of electrodes that show a positive correlation toward electrode #1 as task groups. If so, one would have to consider that task groups span geometrical compartments. At this point one should keep in mind such a possibility but the evidence is currently too weak and needs further research.

### Conclusions

The gastrocnemius muscle is very much structured, contains multiple heads in the proximal regions, and the upper and inner aponeuroses are attached to the Achilles tendon in the distal part. The multiple head structure lead to the assumption that different regions of the muscle must be activated compartment wise. From this work one can conclude that there is a proximal and a distal compartment that can be characterized by a negative correlation or by a phase inversion of the monopolar EMG-currents, which reflects a regional electrical property and is, *per se*, not related to the muscle activation process. In contrast to bipolar EMG-potentials, the raw monopolar EMG-currents of the two compartments are strongly correlated, especially for the calf rising task, which is a more complex movement than balancing in a tiptoe position. Because the muscle fibers do not span the whole length of the muscles one has to conclude that the MUs were synchronized by synchronizing the various motor nerves. This and a previous study show that it is essential to measure monopolar signals and use non-isometric contractions to observe the effects of synchronization of the EMG-signals (von Tscharner, 2014). One could speculate that compartmental differences can, if at all possible, be observed if more complex movements, which generate rotational forces at the knee, are used.

### Conflict of interest statement

The authors declare that the research was conducted in the absence of any commercial or financial relationships that could be construed as a potential conflict of interest.
